# Estimation of parasite age and synchrony status in *Plasmodium falciparum* infections

**DOI:** 10.1038/s41598-020-67817-6

**Published:** 2020-07-02

**Authors:** Laura Ciuffreda, Felix Kwame Zoiku, Neils B. Quashie, Lisa C. Ranford-Cartwright

**Affiliations:** 1 Institute of Infection, Immunity and Inflammation, College of Medical, Veterinary and Life Sciences, University of Glasgow, Glasgow, UK; 2 Centre for Tropical Clinical Pharmacology and Therapeutics, University of Ghana Medical School, Korle Bu, P.O. Box GP4236, Accra, Ghana; 3 Department of Epidemiology Noguchi Memorial Institute for Medical Research, College of Health Sciences, University of Ghana, P.O. Box LG581, Legon, Ghana; 4 Institute of Biodiversity Animal Health and Comparative Medicine, College of Medical, Veterinary and Life Sciences, University of Glasgow, Glasgow, UK

**Keywords:** Parasite biology, Parasitology, Malaria

## Abstract

Human malaria parasites have complex but poorly understood population dynamics inside their human host. In some but not all infections, parasites progress synchronously through the 48 h lifecycle following erythrocyte invasion, such that at any one time there is a limited spread of parasites at a particular time (hours) post-invasion. Patients presenting with older parasites, and with asynchronous infections, have been reported to have higher risks of fatal outcomes, associated with higher parasite biomass and multiplication rates respectively. However, practical tools to assess synchrony and estimate parasite age post-invasion in patient samples are lacking. We have developed a novel method based on three genes differentially expressed over the parasite intra-erythrocytic lifecycle, and applied it to samples from patients with uncomplicated malaria attending two health clinics in Ghana. We found that most patients presented with synchronous infections, and with parasites within 12 h of erythrocyte invasion. Finally we investigated if clinical features such as fever and parasite density could act as predictors of parasite age and synchrony. The new method is a simple and practicable approach to study parasite dynamics in naturally-infected patients, and is a significant improvement on the subjective microscopical methods for parasite staging in vivo, aiding patient management.

## Introduction

Malaria parasites of the species *Plasmodium falciparum* classically progress synchronously through their 48-h lifecycle in the erythrocytes, from invasion to release of new merozoites, which gives rise to the periodic fevers associated with malaria infection^1,2^. However, the extent to which natural infections display a synchronous pattern of growth is unclear; fever patterns, especially in *falciparum* malaria, are often irregular and show no distinct periodicity^3^. Both oscillatory and non-oscillatory patterns in parasite densities were observed in non-immune patients deliberately infected during malaria therapy for neurosyphilis^4,5^. The degree of synchrony is relevant clinically: individuals with asynchronous infections have been reported to have higher parasite multiplication rates, associated with increased disease severity^6,7^, and result in a more rapidly expanding parasite population, which may outstrip antiparasitic host responses, or interventions such as drug treatment^8^.

A parasite infection is defined as synchronous when merogony (schizogony) occurs with a standard deviation of less than 4 h^9^, i.e. 68% of circulating parasites are within a 4 h age window. Using a combination of morphological features to define the “age” [hours post infection (hpi)] of the ring stages seen in circulation, most naturally occurring infections have been described as synchronous^10^, but the presence of two parasite broods shifted by 24 h has also been described in some patients, supported by the observation that parasite-negative samples are rarely seen in infected patients^9^. However, the morphological characteristics used can be influenced by the immune status of the patient^10^, and the distinction between young and old ring-stage parasites can be difficult to assess objectively. Transcriptome analysis has recently been used for the determination of parasite age (hpi) in field isolates^11,12^, defined by the stage-specific expression patterns of the ~ 950 genes transcribed over the ring stage^13^.

What drives synchrony in natural infections is unknown. The asynchronous growth of *P. falciparum *in vitro suggests a role for host factors, such as fever^14^. However, synchronous infections have been observed in asymptomatic individuals^15^, suggesting that fever is not the only determinant for parasite synchrony.

Parasite dynamics in malaria patients may also depend on how the blood-stage infection initiates: a single mosquito bite inoculates 30–50 sporozoites^16,17^, of which one or two successfully infect hepatocytes^18^. Non-simultaneous rupture of the mature infected hepatocytes releases broods of parasites with shifted temporal patterns^19^. Furthermore, infections with multiple clones (genotypes) of the parasite are also very common in endemic areas^20^, and these may originate at different times; an absence of synchrony between genotypes, but not within the same genotype, in multiple clone infections was observed in studies carried out on asymptomatic children^15,21,22^. Moreover, differences in cell cycle length among genotypes could also affect the synchrony of parasites within an infection, especially when two or more clones are present^7,23^.

Generally, studies on parasite synchrony in vivo are hampered by the lack of simple tools able to distinguish between synchronous and asynchronous infections, and to define the age(s) post invasion of ring stages circulating at any one time. Ethical considerations constrain multiple sampling from the same patient to monitor parasite progression directly. Although whole transcriptome data are reliable and accurate^11,12^, the cost and need for subsequent analysis of large datasets hinders their application to large cohort studies in the field.

We present a simple method for determination of ring-stage parasite age post-invasion, coupling predictive linear models with reverse transcription quantitative PCR (RT-qPCR) of three age-specifically transcribed genes of *P. falciparum*. This method was optimised using in vitro samples and then used to define age (hpi) and synchrony status of circulating, ring-stage parasites from Ghanaian patients with uncomplicated malaria. We analysed the relationship of ring-stage parasite age and synchrony status in patient infections with infection parameters including fever, multiple clone infections and parasitaemia, with the aim to identify predictors for ring-stage parasite age and synchrony in malaria infections. Finally, we compared the age of ring-stage parasites in some patient samples defined by the current microscopical approach to the predicted age from the model using transcriptional data.

## Results

### Parasite age-specific genes over the ring stage

**Figure 1:**
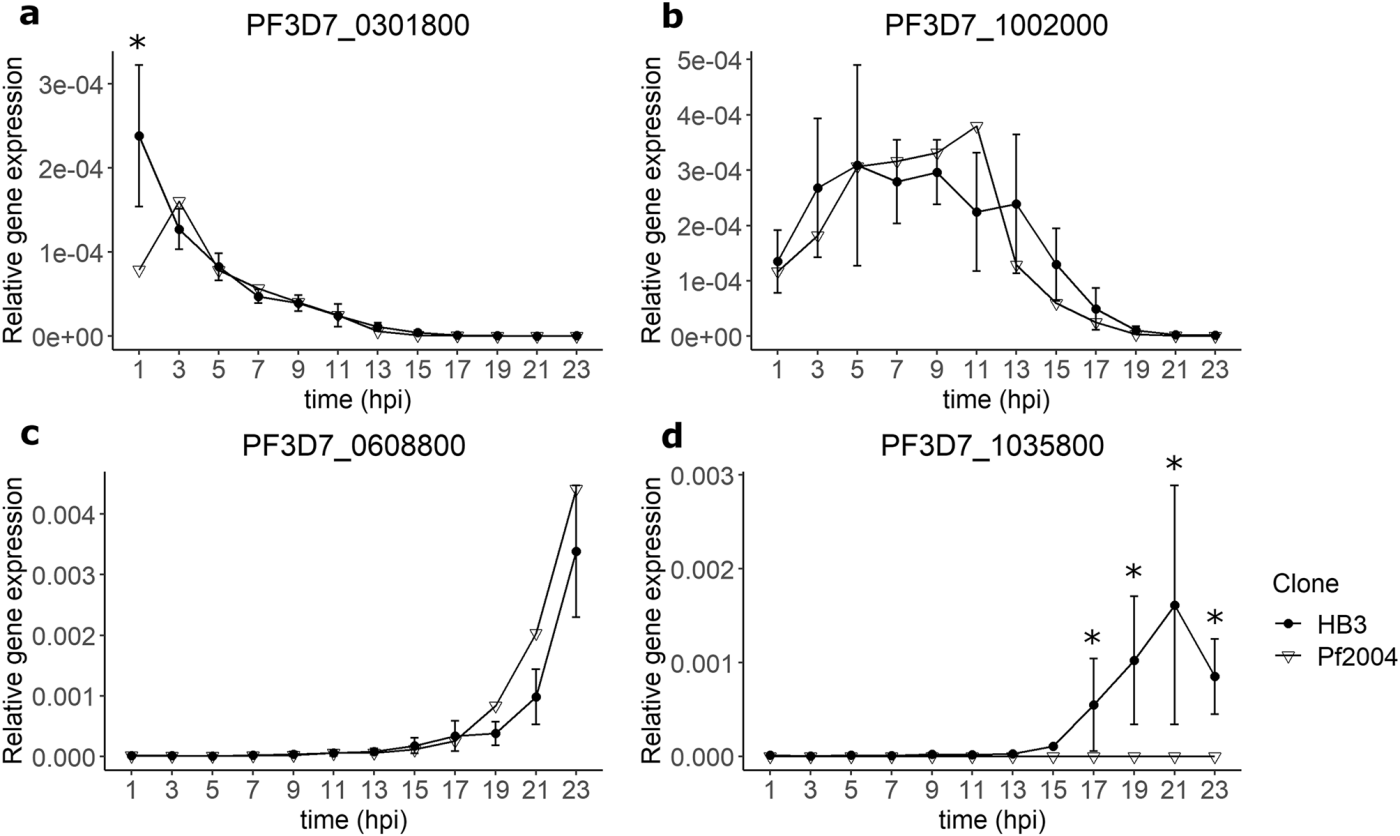
Relative gene expression of *PF3D7_0301800*, *PF3D7_1002000*, *PF3D7_0608800* and *PF3D7_1035800* over time in ring-stage parasites of two *P. falciparum* lines (HB3 and Pf2004). Relative gene expression was calculated as 2^−ΔCt^ (where ΔCt at each time point = ctgenex at time x − ct18S at time x) for PF3D7_0301800 (**a**), PF3D7_1002000 (**b**) and PF3D7_1035800 (**d**) or using the Pfaffl method^51^ for PF3D7_0608800 (**c**). Each point represents the average relative gene expression at a specific time point, while the error bars represent the standard deviation calculated from three replicates for each parasite line (HB3 = black circle, Pf2004 = unfilled inverted triangle). Asterisks show significant difference (one-sample t-test, α = 0.05) between HB3 and Pf2004 lines at each time point.

Published gene expression data^24^ were analysed to select four genes matching either early (*PF3D7_0301800, PF3D7_1002000*) or late (*PF3D7_0608800*, *PF3D7_1035800*) gene expression over the first 24 h post erythrocyte-invasion (ring stage). To assess their validity as parasite age markers in RT-qPCR, their gene expression relative to the reference 18S rRNA was assayed for two parasite lines (HB3 and Pf2004) over the 24 h ring-stage parasite development in highly synchronised cultures. In the HB3 parasite line, *PF3D7_0301800* had the highest relative gene expression in the early ring stages, *PF3D7_1002000* at early-mid ring stage, while *PF3D7_0608800* and *PF3D7_1035800* presented highest relative gene expression at the late ring stage of the parasite. Relative gene expression in the Pf2004 line was similar to that of HB3 for three genes (*PF3D7_0301800, PF3D7_1002000, PF3D7_0608800*) (Fig. 1a–c) while much lower relative expression was observed for *PF3D7_1035800* (Fig. 1d, one-sample t-test, p < 0.05 at time points from 15 to 23 hpi). Assuming that high variability in gene expression in cultured lines would correspond to possible variability in patient samples, *PF3D7_1035800* was excluded from further analysis. A significant difference in relative gene expression between the two parasite lines was also observed at 1 hpi for *PF3D7_0301800* (Fig. 1a, one-sample t-test, p = 0.04), however this gene was retained and the variability taken into account in the model. No significant difference between expression levels in HB3 and Pf2004 was observed for any other gene at any time point. The sensitivity of the RT-qPCR assays was found to be 400 parasites/μL by serial dilution.

### Predictive model for parasite age and synchrony

A GLMM was used to predict the time post-invasion (hpi) of the ring-stage parasites present in a blood sample. Raw Ct values of genes *PF3D7_0301800* (Gene 1), *PF3D7_1002000* (Gene 2) and *PF3D7_0608800* (Gene 3) obtained from the experiments of RT-qPCR described above were used to build the model. The model that best fitted the gene expression data was the most complicated model which included both main effects and interactions of the three genes (Model 1), and the random effect of replicate:

Model 1 $$\begin{aligned} {\text{Parasite age }}\left( {{\text{hpi}}} \right) \, &= {\text{ Ct}}_{{({\text{Gene1}})}} + {\text{ Ct}}_{{({\text{Gene2}})}} + {\text{ Ct}}_{{({\text{Gene3}})}} + \, \left[ {{\text{Ct}}_{{({\text{Gene1}})}} \times {\text{ Ct}}_{{({\text{Gene2}})}} } \right] \, + \, \left[ {{\text{Ct}}_{{({\text{Gene1}})}} \times {\text{ Ct}}_{{({\text{Gene3}})}} } \right] \, \\&\quad+ \, \left[ {{\text{Ct}}_{{({\text{Gene2}})}} \times {\text{ Ct}}_{{({\text{Gene3}})}} } \right] \, + \, \left[ {{\text{Ct}}_{{({\text{Gene1}})}} \times {\text{ Ct}}_{{({\text{Gene2}})}} \times {\text{ Ct}}_{{({\text{Gene3}})}} } \right] \, + \, \left( {{1}|{\text{replicate}}} \right). \end{aligned}$$

The age of parasites was associated significantly with the expression levels (as measured by Ct values in RT-qPCR assays) of the three genes chosen and their interactions (Χ^2^ = 175 (7 d.f.), p = 2.2e−16***). The model explained 96.6% of variance in parasite age.

**Figure 2:**
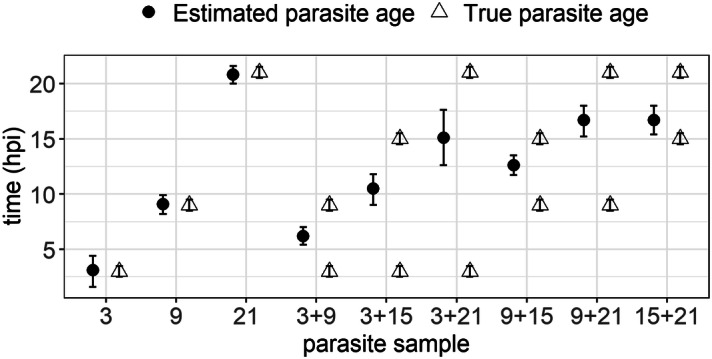
Paired comparison of true and estimated parasite age for pure and mixed-stage samples and corresponding 95% confidence interval (CI). True parasite age (hpi, black circle) are shown in comparison to the estimated parasite age after model prediction (open triangle) in highly synchronous and mixed stage samples. Error bars correspond to 95% CI around the parasite age for the estimated samples from the model, and to the range of age for the true parasite age samples.

Model (1) was then used as a predictive model to infer parasite age in three highly synchronous and six mixed-stage samples in order to assess (i) the accuracy of the predictions of parasite age based on the gene expression levels of the three genes, and (ii) whether the model was able to distinguish between synchronous (parasites of the same ring-stage age) and asynchronous (parasites of mixed ages in the ring stage) samples. The mixed stage samples used to validate the model were mixtures of two time points: 3 + 9, 3 + 15, 3 + 21, 9 + 15, 9 + 21, 15 + 21 hpi. Bootstrapping was used to estimate the predicted parasite age from the model and the prediction error was obtained by retrieving 2.5% and 97.5% percentiles and calculating the confidence interval (CI) width around the median values (Predicted age) (Fig. 2).

Good predictive ability for model (1) was observed in synchronous time points (Fig. 2). However, for samples containing mixed stages, the predicted age corresponded in almost every case to an intermediate point of the two samples present (e.g. for the sample containing a mixture of 3 and 9 hpi parasites, the predicted age was 6.2).

The accuracy of the prediction for age (as indicated by the width of the 95% CI) also correlated with the range of age within the sample: when ring-stage parasites differed widely in age (e.g. a mixture of 3 and 21 hpi), the width of the 95% CI was greater than when the two samples were closer in age (Table 1, Fig. 2). Synchronous or mixed stage samples containing parasites with a known difference in age ≤ 12 hpi had a CI width after prediction of < 3, while the samples with parasites with a difference in age > 12 hpi (3 + 21 hpi) had a CI width > 3. A threshold of CI width = 3 was applied to differentiate between synchronous and asynchronous infections.

**Table 1:** Model validation using synchronous and asynchronous parasite ring-stage samples from culture.

Sample (hpi)	Predicted age (hpi)	CI (2.5–97.5%)	Confidence interval width
3	3.1	1.6–4.4	2.8
9	9.1	8.2–9.9	1.7
21	20.8	20–21.6	1.6
3 + 9	6.2	5.4–7	1.6
3 + 15	10.5	9–11.8	2.8
3 + 21	15.1	12.6–17.6	5
9 + 15	12.6	11.7–13.5	1.8
9 + 21	16.7	15.2–18	2.8
15 + 21	16.7	15.4–18	2.6

Predicted age of parasites of synchronous and asynchronous samples; predicted age corresponds to median values after bootstrapping (n = 1,000); 2.5% and 97.5% percentiles were also obtained. Confidence interval (CI) width was calculated by the formula: [97.5–2.5%] and is an indication of the goodness of the model in predicting the age of that specific sample. For each single time point, three replicates were carried out. The mean Ct for each gene was used as input for the predictive model.

### Patient and infection characteristics

A total of 88 people were enrolled from two collection sites (25 patients from LH and 63 from DHC). Patient characteristics at admission to the study are detailed in Table 2. The only significantly different characteristic between people recruited at the two sampling centres was ethnic group. Most patients reported symptoms (headache, vomiting and diarrhoea) in the previous 2–3 days before attending the hospital. Fever at the time of sampling was common, and most patients presented with mild anaemia. The median parasite density observed in patients was 8,500 parasites/μL of blood (14,896 parasites/μL in LH and 5,160 parasites/μL in DHC), and 64% were single clone infections as defined by PCR.

**Table 2:** Admission characteristics of patients with malaria in two distinct sites in Accra, Ghana.

Demographic and clinical characteristics	Lekma Hospital (LH) n = 25	Danfa Health Centre (DHC) n = 63	Total n = 88	p value^a^
Gender, female (%)	11	37	48 (54.5)	0.24
Ethnic group, Akan (%)	8	21	29 (32.9)	1
Ethnic group, Ga (%)	13	12	25 (28.4)	0.003*
Ethnic group, Ewe (%)	4	17	21 (23.8)	0.4
Ethnic group, others (%)	0	13	13 (14.9)	0.01*
Median patient age, years	15	21	20.5	0.78
Patients < 5 years (%)	4	9	14 (15.9)	1
Patient with fever, body T > 37.5 °C (n, %)	9 (n = 22)	28 (n = 42)	37 (n = 64, 57)	0.06
Mean Hb children, year < 5, g/dL (n, SD)	11.72 (n = 5, 1.7)	10.4 (n = 10, 2.2)	10.8 (n = 15, 2.1)	0.2
Mean Hb children, 6 < year < 14, g/dL (n, SD)	11.7 (n = 6, 2.1)	11.4 (n = 15, 1.6)	11.5 (n = 21, 1.7)	0.7
Mean Hb adults, year > 15, g/dL (n, SD)	13.3 (n = 11, 2.4)	12.3 (n = 32, 2.2)	12.6 (n = 43, 2.3)	0.2
Parasites/μL, median (n, range)	14,896 (n = 20, 379–145,743)	5,160 (n = 54, 40–149,920)	8,500 (n = 74)	0.076
Single clone infections, percentage (n, %)	17 (n = 22, 77.2)	33 (n = 56, 58.9)	50 (n = 78, 64.1)	0.19
Antimalarial use (past 7 days)	3	6	9	1

Demographic and clinical characteristics of patients who took part in the study; values refer to the two sites separately in the first two columns (Lekma Hospital and Danfa Health Centre) and as a whole in the last column, *SD* standard deviation. Ethnic group was self-reported; four low frequency reported ethnicities are grouped as “others”. Body temperature data from 17 patients in DHC were excluded, because they were taken using a malfunctioning forehead body temperature gun thermometer which was routinely used in the clinic at that time.

^a^p value for significance of difference between LH and DHC, tested as described in methods.

### Parasite age and synchrony status in patient samples

**Figure 3:**
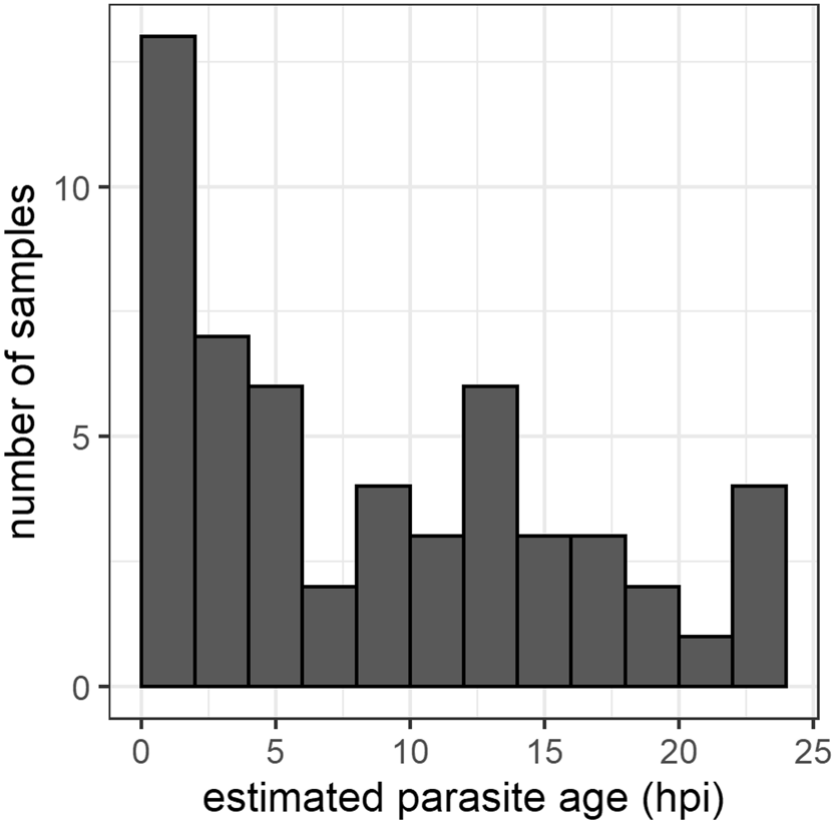
Distribution of parasite age in patient samples, as predicted by Model (1), in samples from two sites in Accra, Ghana. Distribution of predicted age in patient samples collected in LH and DHC in Accra. Predicted age was determined by RT-qPCR analysis of three genes [*PF3D7_0301800* (Gene 1), *PF3D7_1002000* (Gene 2) and *PF3D7_0608800* (Gene 3)] followed by prediction using linear mixed effect model in R (Model 1). Median values obtained after bootstrapping were used as Predicted age. Samples with negative values for predicted age (n = 3) were adjusted to 1 hpi, and those with predicted age > 23 h (n = 2) were adjusted to 23 hpi (maximum parasite age modelled).

Of the 88 patients attending the clinic, nine were excluded because they had taken ACTs before attending the clinic, five because they had a negative result by nested PCR (nPCR) and microscopy, and two because they did not reach the threshold of detection of 400 parasites/μL. As the model prediction was based on the Ct values from three genes, samples which presented amplification by RT-qPCR of only one or two genes were excluded from the analysis. A total of 54 samples remained for further analysis (17 in LH and 37 in DHC). Parasite age and synchrony status of parasites present in these patient samples were estimated using the predictive model and method previously described. The parasite ages predicted from the model are shown in Fig. 3. More than half of patient samples (n = 35, 64.8%) had a predicted age of < 12 hpi, with most of them (48%) with very young parasites (0–5 hpi). Using the strategy based on CI width, it was assessed that the majority (61%, n = 33) of patient samples presented with synchronous infections.

### Predictors of parasite age and synchrony status in patient samples

In the GLM analysis, the only explanatory variable significantly correlated with predicted parasite age was body temperature at admission (df = 1, p = 0.03), but this explained only 6.7% of the variance in predicted parasite age (R^2^ = 0.067). Body temperature (p = 0.03), parasitaemia (p = 0.007), number of clones (p = 0.03) and time of sampling (p = 0.02) were significant explanatory variables for synchrony of infection, and the optimal model explained the 42% of the variance in the probability of synchrony (McFadden R^2^ = 0.42). Synchronous infections were more likely to be found in individuals with higher body temperatures, lower parasitaemia with a single genotype, attending the clinic later in the day (Table 3).

**Table 3:** Predicted effects of significant factors explaining the likelihood of a synchronous infection.

Factor	Unit	Effect on odds of synchronous infection	OR (2.5–97.5% CI)
Body temperature	1 °C increase	120% increase	2.2 (1.1–5.1)
Parasitaemia	1 parasite/μL increase	1% decrease	0.99 (0.99–0.99)
Multiplicity of infection	Multiple genotype infection	85% decrease	0.15 (0.02–0.7)
Time of sampling	1 h later	93% increase	1.93 (1.5–3.7)

### Comparison of parasite age defined by microscopy and gene expression

**Figure 4:**
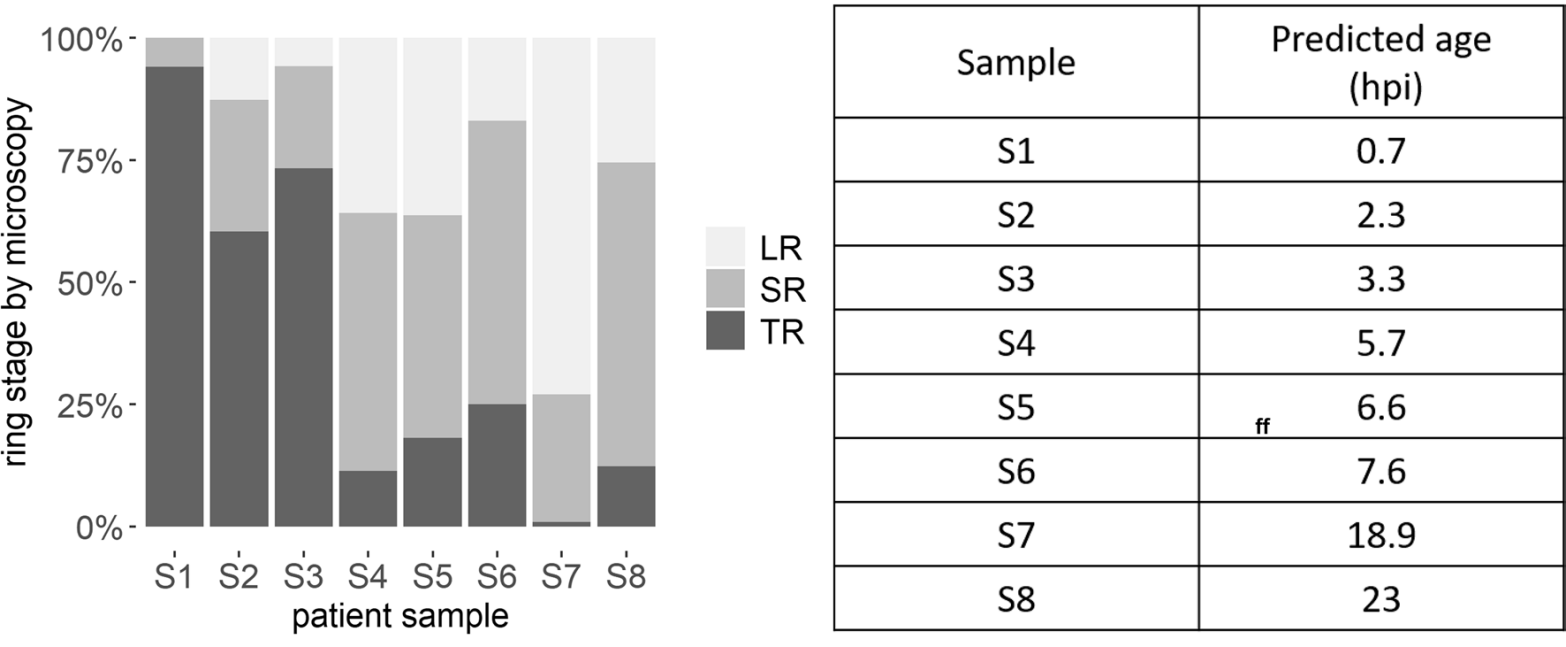
Proportion of ring stage identified by microscopy in eight patient samples from Ghana compared to their parasite age predicted using Model 1. Ring-infected cells observed on Giemsa-stained thin smears were differentiated into three morphological stages using published methodology^25^. A minimum of 50 parasites were counted on each slide. *TR* tiny rings (0–6 hpi), *SR* small rings (6–16 hpi), *LR* large rings (16–24 hpi).

Morphological assignment of the ring-stage parasites by microscopy into three classes, tiny rings (0–6 hpi), small rings (6–16 hpi) and large rings (16–24 hpi)^25^, showed good agreement with the age predicted from the gene expression model (Fig. 4).

## Discussion

We developed a new and simple method to estimate the age (hpi) of circulating *P. falciparum* parasites, based on analysis of three differentially expressed genes over the *P. falciparum* ring developmental stage. The method appears to correlate well with previously published methods to define the age of ring-stage parasites (hpi) based on morphological assessments and microscopy. Using the gene expression method, we were able to determine parasite age and synchrony status in samples from patients with uncomplicated malaria in a field study in Ghana, and identified important predictors of these two variables in natural infections.

A high proportion of samples collected in the study presented early ring-stage parasites (Fig. 3), in agreement with previous whole transcriptome studies of samples from patients in Malawi and Senegal^11,12^, and studies based on microscopy^2^. This observation has been suggested to be linked to the discomfort (e.g. fever) caused by schizogony in uncomplicated malaria patients, which induces people to seek diagnosis and treatment promptly^26^.

Body temperature was the only factor significantly predictive of parasite age in the modelling analysis. Patients with fever at the time of sampling were generally more likely to have young rings, probably because schizogony and the subsequent release of TNF have been demonstrated to cause a transient, short-lived fever^14^. However, the model explained very little (6.7%) of the variance in predicted parasite age, suggesting that other, unknown, factors have a larger influence.

Body temperature, number of clones, parasitaemia and time of sampling were the identified significant predictors for synchrony, with infections in patients with fever, attending the clinic later in the day, presenting with low parasite densities and a single genotype infection more likely to be synchronous. Patients presenting with high body temperatures had more synchronous parasites than those with more normal body temperatures. The effect of fever on specific developmental stages may be responsible for this observation. The exposure of parasites in vitro to high temperatures (39–40 °C) may cause synchronisation^27^ presumably by killing late-stage parasites^14,28^. In this case the infection becomes synchronous, with survival only of the newly invaded parasites derived from the ruptured schizonts that caused the onset of fever^29^.

Parasitaemia was also identified as a significant inverse predictor of synchrony, with lower parasite densities associated with higher likelihood of synchrony. Synchronous infections have been previously associated with lower parasite multiplication rates in non-immune individuals^5^, which could result in lower parasite densities. Furthermore, the release of merozoites induces the host-immune response and triggers fever, which in turn reduces parasite densities, possibly by killing the late stage of the parasite^30,31^. In addition, simulation studies suggest that the host immune response is more likely to reduce parasite densities in highly synchronous infections, by acting against a single stage of the parasite^32^.

Synchrony was also inversely associated with multiplicity of infection: synchronous ring-stage infections were more likely to consist of a single genotype than asynchronous infections. This finding agrees with previous research showing that asynchronous infections were more diverse than synchronous infections^22^. Multiple clone infections can originate from mixed genotypes of sporozoites inoculated in a single mosquito bite^33^, or from different mosquito bites, occurring at different times; in the latter case blood-stage infections would be initiated at different times from separate exo-erythrocytic schizonts. Furthermore, multiple clones may have different cycle lengths and therefore, even if starting at the same time, could proceed asynchronously in vivo^34^.

Time of sampling was also identified as a significant predictor of synchrony, with more synchronous samples collected later in the day. As previously noted, these later infections were more likely to present with fever, which itself was linked to infections with young rings. This finding is however contrary to what is expected if the circadian rhythm (and specifically plasma melatonin concentrations) had an effect on parasite growth^35,36^. Melatonin is a hormone released by the pineal gland which regulates the circadian rhythm. In humans, its release peaks in the middle of the night (2 a.m.–4 a.m.) and gradually decreases in the morning^37^. It has previously been suggested that peaks of melatonin induce accelerated parasite development towards the schizont stage^35^. If this was the case, samples collected earlier in the morning should have younger and more synchronous parasites than those in the afternoon, but this was not observed. It is worth noting that the sampling time in this study was limited by the opening hours of the diagnostic units in the clinics (8 a.m.–2 p.m.). Sampling over different times during day and night will be needed to better understand the relation between circadian rhythm and parasite synchronisation in *P. falciparum* infections in vivo.

In conclusion, here we present a novel method to determine circulating parasite age and synchrony status of parasite populations in samples from patients with *P. falciparum* malaria. We suggest that this method could be used in studies of parasite dynamics in vivo as an alternative to microscopical staging or whole genome transcriptomics, representing a significant improvement over the available methods for parasite staging in vivo. For example, parasite clearance rates are influenced by the age of parasites at the point of artemisinin treatment; young ring-stage parasites (3–9 h post invasion) are hyper-susceptible to artemisinins^38,39^, whereas resistance to artemisinin manifests as a loss of susceptibility in older ring-stage parasites (9–20 h post-invasion)^39^. Therefore knowing the age of parasites at the point of treatment is important to interpret clearance rates and to identify potential artemisinin-resistant cases. Furthermore, the parasite stage of development is important for patient prognosis, especially in severe malaria: the presence of older rings implies a higher load of parasites sequestered, which is associated with fatal outcomes^10,26^. The prompt identification of those patients having an infection enriched with older ring-stage parasites may help adequate patient management in malaria endemic countries. A limitation of the predictive model is that parasite broods need to be more than 12 h different in median age (hpi) to be identified as asynchronous. In future, the use of expression data from more than three genes for parasite staging could offer a better resolution in parasite age. Future studies may focus on comparing parasite age and synchrony status in patients presenting different clinical manifestations of the disease, such as asymptomatics or patients with severe malaria.

## Methods

### Synchronisation of *P. falciparum *in vitro*,* and preparation of RNA and cDNA samples

*Plasmodium falciparum* parasites were grown in culture using standard methodology^40^. Parasite lines HB3 (Honduras) and Pf2004 (Ghana) were used for all synchronisation experiments. Briefly, parasites were treated with sorbitol^41^ 48 h and 36 h before the experiment to obtain a culture enriched with schizonts. Parasites were then tightly synchronised using a previously published protocol^42^ to obtain parasites within a window of invasion of 1 h. This ring-stage culture [1 h post invasion (hpi)] was then grown on for 24 h, with regular removal of 3 mL volumes, collected every 2 h. These samples were washed twice in PBS and the pellets stored at − 80 °C until RNA extraction. Parasitaemia and parasite stages present in each culture were assessed by thin blood smear and microscopy identification at each sampling timepoint. Three biological replicates were performed, each from a different parasite culture. RNA was extracted using the RNAeasy kit (Qiagen) following the manufacturer’s instructions, and was eluted in 30 μL of RNase–free H_2_O, and quantified by Nanodrop. All samples were treated with DNaseI (Invitrogen; 1 unit/μg RNA) to remove contaminating genomic DNA. cDNA was generated using M-MLV reverse transcriptase (Invitrogen) and primed with random hexamers following the manufacturer’s instructions.

### Identification of gene markers to define parasite age post-erythrocyte invasion

Genes showing specific expression patterns throughout the ring stages of development (1–6 hpi, 12–18 hpi, 19–24 hpi, 12–24 hpi) were identified from previously published microarray data of *P. falciparum* intraerythrocytic stages^24^ using Pearson’s correlation coefficient analysis. The specificity of timing of expression was confirmed by RT-qPCR analysis using the synchronised culture samples, using gene-specific primers (Supplementary Table S1) and relative gene expression was measured using 18S rRNA (A type: *PF3D7_0531600*, *PF3D7_0725600*) as the reference gene^43^*.* The sensitivity of the RT-qPCR was determined by serial dilution of a culture of synchronised parasites, at known parasitaemia, with uninfected RBCs, with the threshold of detection defined as the parasitaemia of the last diluted sample at which amplification occurred. All reactions were performed using an Applied Biosystem 7,500 Real time PCR machine. A Ct value higher than 35 was considered as negative.

### Validation of the predictive model to predict asynchronous infections

Equal quantities of cDNA from pairs of synchronous samples separated in time (5 mixtures of 3 + 9 hpi, 9 + 15 hpi, 15 + 21 hpi, 3 + 21 hpi, 9 + 21 hpi) were mixed together, and then amplified by RT-qPCR before model prediction.

### Study site, ethical approval and patient recruitment for malaria patient sampling

Ethical approval for the study was obtained from the Noguchi Memorial Institute for Medical Research/University of Ghana Institutional Review Board (study number NMIMR.IRB CPN 107116-17), the Ghanaian Science and Technology Board, (NMIMR STC Number: 3(6) 1016-17), and the University of Glasgow MVLS College Ethics Committee (project number: 200160164). The research was conducted in accordance with the relevant regulations and guidelines.

Patients were recruited to participate in the study from two sites in Accra, Ghana: Lekma Hospital (LH) in the urban district of Ledzekulu-Krowor, and Danfa Health Centre (DHC) situated in the peri-urban La Nkwantanang Madina Municipal district. The Greater Accra region is hypo-endemic for malaria with parasite prevalence in children of 2–10 years of age of 1–10%^44^ and malaria transmission estimated at < 50 bites/person/year^45^. Fully-informed consent was obtained from each of the patients. For individuals below the age of consent (18 years old), consent was obtained from their guardians, with assent obtained from the individual for those 12–17 years old. Inclusion criteria to take part in the study were the presence of mono-infection with *P. falciparum* as detected by microscopy, and the absence of severe manifestations of the disease.

### Collection and processing of patient blood samples

For each patient, age, gender, ethnic group, axillary (armpit) body temperature, haemoglobin (Hb) levels (Hb/L), time of sampling, and parasite density (parasites/μL blood) were recorded. Parasite densities were counted by thick blood smears, while Hb levels were obtained by the Abacus 301 system present in the clinics or directly using a Hemocue Hb 201 + system.

Samples of patient blood for both parasite RNA and DNA analysis were collected. For the RNA analysis, a maximum of 300 μL of intravenous blood was collected in a Sarstedt microvette CB300 μL, (K_2_ EDTA) and immediately snap-frozen on dry ice to preserve the RNA. RNA was extracted, cDNA synthesised, and RT-qPCR performed as described for in vitro grown parasites, to define the parasite age (hpi) present in each sample.

Multiplicity of infection was assessed by PCR amplification of genes *MSP1 *(*PF3D7_0930300*), *MSP2 *(*PF3D7_0206900*) and *GLURP* (*PF3D7_1035300*) following published methods^46^, using genomic DNA extracted from ~ 20 μL blood spotted onto a Whatman filter paper^47^.

### Prediction of parasite age

A generalised linear mixed effect model (GLMM) procedure was used to build a predictive model for parasite age and synchrony using the synchronised culture material. The fixed effect explanatory variables were the mean cycle threshold (Ct) values of each of the technical replicates obtained from RT-qPCR for the chosen genes. Biological replicate was included as a random effect. The time post-invasion of the culture sample (parasite age—ranging from 1 to 23 hpi) was used as the outcome variable of the model. A stepwise backward selection^48^ was used to identify the significant fixed effects, and likelihood ratio tests were used to compare the different models. A Shapiro–Wilk test was adopted to assess the normality of model residuals. Once the optimal minimal model was identified, prediction of unknown age of parasites in in vitro and patient samples was carried out by bootstrapping, with the median set as parasite age. Package lme4^49^ in R software (version 3.4.1)^50^ was used for model building.

### Statistical analysis

A one-sample t-test was used to determine the statistical significance of the difference between relative gene expression of HB3 and Pf2004 lines in culture. Student’s t-test (when data were normally distributed) and non-parametric tests (when data were not normally distributed) were adopted to assess the statistical difference between the clinical parameters in patient samples collected at the LH and DHC sites. Generalised linear models (GLM) were used to determine the relationship between parasite age or synchrony and other clinical and non-clinical parameters (gender, ethnic group, body temperature, time of sampling, parasitaemia, Hb level, presence of single (one genotype) or multiple (> one genotype) infections). A GLM with gamma family was used when parasite age was the independent variable, while a logistic regression model was used when synchrony was the independent variable. Data were analysed using R software (version 3.4.1)^50^.

### Definition of parasite age by microscopy

For eight patients selected to represent a range of predicted parasite ages in synchronous infections, Giemsa-stained thin smears were examined microscopically and the parasites present were assigned to three morphological stages using published criteria^25^. For each sample, at least 50 parasites were evaluated with the microscopist blinded as to the predicted parasite age from the model.

## Supplementary information

Supplementary file1

## Data Availability

All data generated in this work is available in the main text and supplementary information of the manuscript.
