# Supplementary material for: Estimation of parasite age and synchrony status in Plasmodium falciparum infections
Source: Sci Rep. 2020 Jul 2;10:10925. doi: 10.1038/s41598-020-67817-6 (PMC7331735; doi:10.1038/s41598-020-67817-6)
Supplement: Supplementary file 1 — Supplementary file1 [file 41598_2020_67817_MOESM1_ESM.pdf]

Supplementary material

Estimation of parasite age and synchrony status in *Plasmodium falciparum* infections

Laura Ciuffreda[1], Felix Kwame Zoiku[3], Neils B. Quashie[2,3], Lisa C. Ranford-Cartwright[1,4]*

[1] Institute of Infection, Immunity & Inflammation, College of Medical, Veterinary and Life Sciences, University of Glasgow, Glasgow, UK

[2] Centre for Tropical Clinical Pharmacology and Therapeutics, University of Ghana Medical School, P.O. Box GP4236, Korle Bu, Accra, Ghana

[3] Department of Epidemiology Noguchi Memorial Institute for Medical Research, College of Health Sciences, University of Ghana, P.O. Box LG581, Legon, Ghana

[4] Institute of Biodiversity Animal Health & Comparative Medicine, College of Medical, Veterinary and Life Sciences, University of Glasgow, Glasgow, UK

*Corresponding author: Lisa Ranford-Cartwright: lisa.ranford-cartwright@glasgow.ac.uk

Supplementary Table S1: Primers used in the RT-qPCR experiments

18sQ1_For	AGCGGCGAGTACACTATATTCT
18sQ1_Rev	TTAGTAGAACAGGGAAAAGGAT
PF3D7_1002000_For	GCAGAACCAATTTTCGGATTTGA
PF3D7_1002000_Rev	TCACACTTTTCACGTTTGTCACA
PF3D7_0301800_For	TGAAGCAGTTGGTGGAGAAA
PF3D7_0301800_Rev	TGCCATTCAGCTGACACTCT
PF3D7_0608800_For	ACCCAAATGTCTGTGCCTTT
PF3D7_0608800_Rev	CCGGTTCTTCCTAAACCTGTT
PF3D7_1035800_For	AGTGGTACTGAAGGTGATGGA
PF3D7_1035800_Rev	TGTTCTGCATCACTCAAAGCA
